# Highlights from the 58th meeting of the American Society of Haematology, 1–6 December 2016, San Diego, USA

**DOI:** 10.3332/ecancer.2017.725

**Published:** 2017-03-09

**Authors:** Luca Mazzarella

**Affiliations:** European Institute of Oncology, Via Ripamonti 435, Milan 20141, Italy

**Keywords:** leukaemia, lymphoma, minimal residual disease, sequencing, clinical trial, CAR T cell

## Abstract

The recent 58th Annual American Society of Haematology (ASH) meeting held in San Diego shed light on the usual mixture of groundbreaking basic and translational science and the recent practice-changing clinical trials. Recurrent themes this year were the use of recent next-generation sequencing (NGS) techniques to perfect prognostic stratification and disease monitoring. Newer prospects on the role of metabolism in normal and malignant haemopoiesis and mature data on long-awaited trials on immunotherapy and CAR-T cells in lymphoid neoplasms were also discussed.

## Basic and translational science

### Next-generation sequencing-based disease stratification and monitoring

Arguably the hottest topic based on number of presentation was the use of novel sequencing techniques for upfront risk stratification and monitoring of minimal residual disease (MRD). Therapy-tailoring approaches based on MRD measurement were pioneered in chronic myelogenous leukaemia (CML), in the field of acute myelogenous leukaemia (AML), and in the context of acute promyelocytic leukaemia (APL) [1]. These diseases shared the distinctive feature of being associated with specific translocations in the vast majority of cases that provide well defined molecular markers that can be detected by polymerase chain reaction (PCR). On the other hand, application to non-APL AMLs was limited given the underlying genetic heterogeneity. In this setting, flow cytometry-based measurements have played a major role, i.e. trading off applicability for sensitivity. The possibility to sequence multiple genomic regions using NGS now allows, in principle, to conduct patient-specific MRD monitoring. However, the issue is complicated as the genetic identity of the relapsing clone which ultimately affects disease outcome is not univocally understood. If the relapsing clone contains the same mutations as the original clone, then these mutations can be used to track MRD. If instead the mutational landscape changes radically at relapse, then this is not possible. This basic biological issue, which needs to be addressed before one tackles MRD, was studied by several groups (#959 in MDS->AML evolution, #599 in AML relapse post induction chemotherapy). Collectively, these studies suggest the existence of at least two models for clonal evolution upon chemotherapy: some mutations tend to reappear at relapse, some are clearly *de novo* acquired with signatures indicative of a chemotherapy-induced DNA damage mechanism (thus, they are likely to be completely absent and not simply unfrequent at diagnosis). Unfortunately, neither mutation class is ideal *per se* for MRD. The persistent mutations seem to pre-date leukaemia development and as such are not specific enough (persistence upon macroscopic remission does not imply subsequent relapse). The *de novo* mutations clearly cannot be tracked as they are absent at diagnosis. An alternative approach would be to simultaneously track multiple mutations along treatment which, however, would require improvements in detection sensitivity of NGS. It is currently considered to hit a threshold at about 5% variant allele frequency (VAF) which is vastly higher than that required for MRD. A step in this direction is the study conducted by Hirsch *et al* (#1208) who retrospectively analysed 69 consecutive AML patients at diagnosis with a 122 gene sequencing panel and cytogenetics. He identified at least one genetic alteration in 68/69 patients (median = 4) and then monitored MRD in subsequent samplings by constructing patient-specific sequencing panels and applying high sensitivity barcoding-based technology (for a technical reference [2]) which allowed to bring VAF sensitivity down to 0.2%. Unsurprisingly, because of the genetic instability highlighted above, persistence of individual mutations was not significantly associated with outcome (p = 0.08); however, in patients with three or more identified events, the persistence of three or more markers after one course was associated with a very high risk of relapse and lower overall survival (OS). Basic studies suggested that genetic persistence upon treatment of mutations are typically associated with clonal haemopoiesis (CHIP) and is not an intrinsic increased fitness, but it is the conferred ability to tolerate higher DNA damage leading to faster acquisition of new mutations (#958).

Number and type of mutations are also correlated with upfront risk stratification (#286). This has also recently been remarked in the updated European Leukaemia Net recommendations [3], suggesting that genetic information obtainable through NGS is going to be increasingly included in disease classification. Similarly, in Myelodysplastic Syndrome (MDS) Lindsley *et al* (#69) performed the largest retrospective evaluation of the relationship between transplant (including matched or unmatched donors and cord blood) outcome and gene alterations to date. He analysed a set of 127 genes on a consecutive series of 1514 patients. TP53 mutations were strongly predictive of poor OS (eight months if positive) and shorter time to relapse, whereas RAS pathway mutations correlated with shorter time to relapse only, and JAK2 mutations correlated with transplant-related mortality. The authors then integrated genomic and clinical parameters and proposed a new risk stratification with six prognostic groups.

In lymphoid malignances, clonality of T- or B- cell receptors can be used as a molecular marker for MRD. Technological improvements in the last couple of years were presented, and we see now we have several established platforms for this. This year we saw data that consolidate the role of clonality assessment to monitor disease. In particular, MRD negativity measured with the ClonoSEQ assay was shown to correlate very strongly with better progression-free survival (PFS) in relapsed/refractory multiple myeloma (#246). It was also shown in an a posteriori analysis of the CASTOR and POLLUX trials in which the addition of the anti-CD38 monoclonal antibody daratumumab to either lenalidomide/dexamethasone (POLLUX) or bortezomib/dexamethasone (CASTOR) were studied [4, 5].

### Metabolism of normal and malignant haematopoietic stem cells

Leaving the fields of sequencing and MRD, very intriguing data were presented on the hot topic of metabolic control of normal and leukaemic stem cells.

Data from the Trumpp lab (#LBA-4) suggest that numerous metabolic pathways are progressively upregulated upon exit of haematopoietic stem cells (HSC) from dormancy. It is also noted that on the contrary the retinoic acid metabolism is downregulated and actively contributes to HSC quiescence and persistence of the HSC pool in situations of systemic inflammation that normally lead to HSC depletion. As retinoic acid is a vitamin A derivative. The HSC dynamics could be experimentally modulated in mice by diet alone. Even more intriguing were data on mitochondrial exchange dynamics between HSCs and bone marrow stromal cells (BMSC) presented by dr Golan from Tsvee Lapidot's lab (#5). The authors used mice in which mitochondria were engineered to express Green Fluorescent Protein (GFP)-tagged mitochondrial proteins. They transplanted GFP-tagged HSCs into wild type (WT) mice or WT HSCs into GFP-tagged mice and measured GFP in the host HSC or BMSC. To better evaluate the molecular mechanisms ,they also performed *in vitro* experiments with HSC and BMSC. They demonstrate contact-dependent mitochondrial transfer that could be modulated by the activity of the key metabolic regulator AMPK. The flux was bidirectional, but HSCs donated more mitochondria than they acquired. The authors proposed that this is part of a system to maintain oxygen radical levels below a damaging threshold which would impair long-term efficiency of HSCs. Conversely bone formation is also affected by mitochondrial transfer thereby confirming the intimate relationship between HSCs and their stromal niche.

Collectively, the studies illustrated above suggest a key role for metabolism in controlling HSC dynamics. Future research will elucidate whether this layer of control is also at play in malignant stem cells and can be targeted pharmaceutically.

## Myeloid neoplasms-clinical research

### AML targeted therapies

Early phase clinical data on some interesting new drugs were introduced in the difficult setting of AML induction/consolidation therapy: phase 1 trials. This showed the feasibility of adding vadastuximab talirine, an anti CD33 antibody, conjugated with a DNA-crosslinking agent (#211) and Selinexor which is an exportin 1 inhibitor (#212) to standard induction chemotherapy regimens. Preliminary phase 2 data were shown on SL-401, an inhibitor of the IL3 receptor (CD123), in AML patients achieving complete remission (CR) after induction (#215). All these drugs have in common a relatively limited toxicity profile and an expected efficacy on leukaemia stem cells (LSC) [6–8] which might have dramatic impact on relapse rate after induction. The vadastuximab trial was a phase 1b escalating the experimental drug in first line induction therapy in combination with standard 3 + 7 chemo with 42 patients enriched for intermediate (40%) or adverse (43%) MRC risk treated so far. All patients had G4 myelosuppression, and so dos- limiting toxicity (DLT) was defined in terms of days to recovery to acceptable blood counts. A maximum tolerated dose (MTD) was identified at 20 mcg/kg. Non-haematological toxicity was found tolerable. It was further characterised by gastrointestinal symptom and when a good CR+CRi (CRc) rate of 78% was achieved. This is a good result in this high-risk enriched population. The Selinexor trial was also a phase 1b but this time in combination with less conventional Hi dose Ara-C + mitoxantrone induction therapy. Population included first-line and relapsed-refractory (33%) AMLs. Toxicity and efficacy results were less flattering in this case with again 100% G4 haematological toxicity rate, and a more severe non-haematological toxicity (including thromboses and cardiac and cerebellar events). More importantly it had a response rate of 60% which is little far from the 55% achieved with HiDAC/Mito alone. Lastly, the SL-401 trial was the smallest of the three but the intriguing aspect was its focus in the phase 2 expansion stage on MRD+ patients. Only three patients have been treated at the MTD, but the toxicity profile was very limited and initial signs of efficacy (fall in cells expressing stem cell markers) were just observed.

FLT3-mutated AML continues to generate interest as a highly malignant disease driven by a targetable mutation. Last year we saw the first positive phase 3 results with a FLT3 inhibitor (the RATIFY trial with the rather unspecific midostaurin which is currently undergoing Food and Drug Administration (FDA) priority review for FLT3 mutated AML and systemic mastocytosis). Although RATIFY showed better OS compared to placebo, but in absolute terms the results were far from flattering with only 7% improvement in five year OS to 50% [9]. This year we saw final results of the phase 1/2 CHRYSALIS trial with the FLT3 inhibitor gilteritinib (ASP2215) which is a more potent and specific FLT3 inhibitor in patients with relapsed/refractory AML (#1069). Presence of a FLT3 mutation was an inclusion criterium only for expansion cohorts, so that in the end 194/252 patients had a confirmed mutation. Patients were heavily pretreated with multiple lines of therapy including 29% prior stem cell transplant and 25% prior tyrosine kinase inhibitor (TKI) treatment which most commonly was sorafenib. Objective response rate (ORR) in FLT3-mutated patients was 49%, higher than in FLT3wt patients (12%) therebyconfirming target specificity. Responses were slow as expected (7.2 weeks median). Median OS was 31weeks. Overall, these results compare favourable with historical controls, but of course they will need to be confirmed in randomised trials to establish the role of gilteritinib in FLT3-mutated AML.

### When to stop TKI in CML

In CML, the discussion was centred around the long-standing issue of the identification of criteria to discontinue TKI after achievement of molecular response (MR). Dr Mahon (#787) presented results of the EURO-SKI trial which is the largest to date aimed at solving exactly this issue (n = 821 from 11 European countries). Most conventional prognostic factors showed no significant association with MR rate at six months after treatment stop (age, gender, depth of molecular response, or any variable part of the Sokal, EURO, EUTOS, or ELTS scores). Instead, predictive factors were treatment duration and MR duration prior to stop (p < 0.001). Every additional year of treatment increases the odds to stay in MR at six months by 16%. Overall, 52% of the patients showed no disease relapse at 18 months. Interestingly, 80% of relapses occurred within the first six months, so most patients requiring treatment restart can be identified early after stop.

The smaller DESTINY study (#938), conducted in the UK on 174 patients, tested a different approach in which TKI was cut to half dosage for the first 12 months and then stopped. Overall, only 6.9% patients relapsed during the de-escalation phase wherein the time, depth of molecular response at baseline were correlated with relapse rate (18.4% for MR3 versus only 2.4% for MR4).

Globally, these studies lend further support to the strategy of discontinuing and/or de-escalating TKI treatment after prolonged molecular responses which might also have important impact on financial issues and patient quality of life (QOL).

## Lymphoid neoplasms-clinical research

### Lymphoma-controversial data on obinutuzumab

Much debates were generated by the results of the various trials with obinutuzumab (GAZYVA), a glycoengineered type II anti-CD20 monoclonal antibody agent with enhanced direct and antibody-dependent cellular cytotoxicity compared with rituximab. Both these antibodies are manifactured by Roche where they conducted the two phase 3 trials presented at ASH in follicular lymphoma (FL, GALLIUM trial) and diffuse large B cell lymphoma (DLBCL, GOYA trial) respectively. In both trials, the design consisted in randomising chemotherapy-naive patients to receiving either rituximab or obinutuzumab on top of chemotherapy (limited to CHOP in GOYA and including CHOP, CVP or bendamustine according to investigator's choice in GALLIUM). A significant difference was that in GALLIUM, antibodies were maintained in respondents beyond the end of the induction phase with chemotherapy whereas no maintenance was applied in GOYA. Perhaps because of this difference in dose intensity, results were also different. GOYA showed no statistically significant difference whereas in GALLIUM the PFS (the primary endpoint) was statistically different with a three-year PFS of 80% (95% CI 75.9%– 83.6% versus 73.3% (68.8%–77.2%) in favour of orituzumab. These result are likely to spark some controversy, since the modest 7% benefit in PFS needs to be weighed against an equal 7% increase in grade 3 or more toxicity rate (67.8 versus 74.6%) leaving aside financial considerations.

### Lymphomas-multicentric CAR T cell trial

On the CAR T cell front, a field that has much animated debates in the last few ASH editions, a key presentation was the #LBA-6 results which was a prespecified interim analysis from the ZUMA-1 trial on refractory DLBCL. This was the first of two expansion cohorts (the other is on primary mediastinal B-cell lymphoma and transformed follicular lymphoma, not presented). Patients received a target dose of 2 × 10^6^ anti-CD19 CAR T cells/kg after a low-dose conditioning regimen of cyclophosphamide (500 mg/m^2^) and fludarabine (30 mg/m^2^). Trials with CAR T cells often yield impressively high ORR. It was 76% in the 51 patients available for analysis presented here. Perhaps the most striking feature was that CAR T cell therapy could be administered in 101/111 patients with a reasonable median turnaround time of 17 days. Despite the trial being a large multicentric (22 institutions) with centralised cell preparation, it demonstrated that logistical considerations are not an intrinsic limit of the CAR approach and that large scale trials are still feasible.

### Lymphomas-combined DNA/RNA-based stratification?

Staying on lymphoma field, researchers from Duke University (#1087) showed results on the largest analysis of combined whole exome and transcriptome sequencing to date, correlating molecular with outcome data. A multivariate model based on a combination of DNA and RNA-based parameters was able to stratify outcome much better compared to cell of origin-based (activated B-cell (ABC) versus activated centre B-cell (GCB)) or more conventional International Prognostic Index (IPI) score-based stratification. These results may pave the way for a new DLBCL stratification system

### Myeloma, upfront therapy-two not always better than one

Important results regarding the choice of upfront therapy for multiple myeloma (MM) were presented as late-breaking abstract (#LBA1). In the era of post-transplant lenalidomide maintenance, the advantage of additional therapy is unclear. In the StaMINA trial all patients (n = 758) received upfront melphalan + auto transplant and were then randomised to receiving consolidation with lenalidomide, dexamethasone, and bortezomib (RVD) for four cycles, or a second transplant in tandem, or nothing. All three arms subsequently received maintenance lenalidomide until progression. The patient population was well representative with 24% classified as high risk. Overall, no treatment appeared to be statistically significantly superior to a single transplant + lenalidomide in terms of OS nor PFS. Thus, this should be considered the standard of care, although additional research is required to understand if specific patient groups such as those with high-risk cytogenetics would benefit from treatment intensification. On the other hand, as induction therapy was not homogeneous, additional research is needed in order to evaluate if some suboptimal induction regimens would benefit from treatment intensification.

### Graft versus host disease-ibrutinib enters the stage

The last topic selected from this year's abstracts is the final results of the phase 2 study evaluating ibrutinib. This is an oral Bruton's kinase inhibitor approved for CLL and lymphoma in a totally new setting which is of chronic GvHD and refractory to multiple lines of steroid therapy (#LBA-3). Patients who had received between 1 and 3 lines of corticosteroids, with or without additional immunosuppressants; GVHD had been present for a median 13.7 months at study entry. After a median follow-up of 13.9 months, the ORR (based on the 2005 NIH consensus response criteria [10]) was 67% with many durable responses. Interestingly, blood levels of multiple cytokines implicated in cGvHD pathogenesis were reduced significantly in responders, suggesting that ibrutinib is acting on underlying molecular mechanisms and not simply as a symptom reliever. Ibrutinib was granted FDA breakthrough therapy designation for this indication based on preliminary results. Therefore, we see that ibrutinib enters the armamentarium of drugs to be used in this kind of clinically relevant setting.
